# Left ventricle remodeling predicts the recurrence of ventricular tachyarrhythmias in implantable cardioverter defibrillator recipients for secondary prevention

**DOI:** 10.1186/s12872-016-0416-y

**Published:** 2016-11-21

**Authors:** Wei-Chieh Lee, Huang-Chung Chen, Yung-Lung Chen, Tzu-Hsien Tsai, Kuo-Li Pan, Yu-Sheng Lin, Mien-Cheng Chen

**Affiliations:** 1 Division of Cardiology, Department of Internal Medicine, Kaohsiung Chang Gung Memorial Hospital, Chang Gung University College of Medicine, 123 Ta Pei Road, Niao Sung District, Kaohsiung City, 83301 Taiwan; 2 Division of Cardiology, Chang Gung Memorial Hospital, Chiayi, Taiwan

**Keywords:** Implantable cardioverter defibrillator, Ventricular remodeling, Ventricular tachycardia

## Abstract

**Background:**

Implantable cardioverter defibrillator (ICD) is an effective treatment for secondary prevention of ventricular tachycardia/ventricular fibrillation (VT/VF). Left ventricular (LV) remodeling may develop before ICD implant and over time. However, it remains unclear how LV remodeling affects subsequent risk for recurrence VT/VF in ICD recipients under optimal medical therapy.

**Methods:**

From May of 2004 to June of 2015, 144 patients received ICD implantation for secondary prevention were enrolled in this study. All information interrogated from ICD devices during follow-up or ICD therapy history (anti-tachycardia pacing and shock therapy) were reviewed and validated the occurrences of VT/VF.

**Results:**

At a mean follow-up of 1110.5 ± 860.6 days, 53 patients (36.8%) had recurrence of VT/VF episodes and 91 patients had no recurrence of VT/VF episode after ICD implant. Left ventricular end-diastolic volume (LVEDV) > 163.5 mL had significant predictive value for VT/VF recurrence (area under the curve: 0.602, *p* = 0.041). Moreover, the percentage of patients with LVEDV >163.5 mL was significantly higher in patients with recurrent VT/VF than patients without recurrent VT/VF (62.3 vs 40.0%, *p* = 0.010). Left ventricular ejection fraction ≤ 30% (*p* = 0.031), LVEDV > 163.5 mL (*p* = 0.012) and QRS width > 125 msec (*p* = 0.049) were significant predictors for VT/VF recurrence by univariate Cox regression analysis. However, only LVEDV > 163.5 mL (hazard ratio: 2.549, 95% confidence interval: 1.249 ~ 5.201, *p* = 0.010) and QRS width > 125 msec (hazard ratio: 2.173, 95% confidence interval: 1.030 ~ 4.586, *p* = 0.042) were independent predictors for recurrence of VT/VF after multivariable adjustment.

**Conclusion:**

LV remodeling and QRS width > 125 msec were independent predictors for VT/VF recurrence in secondary prevention ICD recipients under optimal medical therapy, independent of LV ejection fraction.

## Background

Implantable cardioverter defibrillator (ICD) has been confirmed to be an effective treatment for selected patients who are survivors of sustained ventricular tachycardia/ventricular fibrillation (VT/VF) unrelated to transient correctable causes or are at risk of sudden cardiac death [1]. Several studies have consistently confirmed the preponderance of ICD therapy over anti-arrhythmic therapy in terms of primary or secondary prevention of sudden cardiac death and mortality in patients with heart failure and reduced left ventricular (LV) function [2–7]. However, ICD therapy is an expensive therapy and is reimbursed only for secondary prevention in many developing countries. Additionally, some ICD recipients for primary or secondary prevention may never experience recurrence of VT/VF and shock therapy during follow-up under optimal medical therapy, including ß–blockers and renin-angiotensin-aldosterone antagonists, and modification of risk factors. Therefore, clinical predictors for recurrent VT/VF as the guidance for ICD replacement are requisite for limited healthcare resources in many developing countries. The majority of studies of clinical predictors for recurrent VT/VF enrolled both primary and secondary prevention ICD recipients. Klein et al. reported that LV ejection fraction < 40%, permanent atrial fibrillation and QRS duration > 150 msec are independent predictors for VT/VF recurrence in ICD recipients for primary and secondary prevention [8]. However, there are limited reports regarding clinical predictors for recurrent VT/VF in patients received ICD only for secondary prevention. Interestingly, Freedberg et al. reported no predictor for subsequent ICD therapy in patients receiving ICD for symptomatic VT or cardiac arrest [9].

Left ventricular remodeling associated with underling heart disease may develop before ICD implant and over time after implant. Moreover, ß–blockers and renin-angiotensin-aldosterone antagonists have been shown to inhibit or reverse LV remodeling rather than changes in LV ejection fraction [10]. However, it remains unclear how LV remodeling affects subsequent risk for recurrence VT/VF in ICD recipients for secondary prevention. Accordingly, we conducted this study to investigate the role of LV remodeling as a predictor for the recurrence of VT/VF in ICD recipients for secondary prevention under optimal medical therapy.

## Methods

### Patient population

From May of 2004 to June of 2015, 144 patients received ICD implantation for secondary prevention were enrolled in this study. All information interrogated from ICD devices during follow-up or ICD therapy history (anti-tachycardia pacing and shock therapy) were reviewed and validated the occurrences of VT/VF and ICD therapy. Ninety-one patients who did not have any VT/VF episode or ICD therapy during follow-up after ICD implant were defined as the “no recurrent VT/VF group”, whereas 53 patients who had experienced VT/VF episodes and appropriate ICD therapies during follow-up after implant were defined as the “recurrent VT/VF group”.

### Definitions

Left ventricular diastolic dysfunction was categorized into 3 stages: stage I (impaired relaxation), stage II (pseudonormalization) and stage III (restrictive filling pattern) according to the echocardiographic patterns of mitral inflow velocity, mitral Doppler tissue imaging of mitral annular motion and pulmonary venous flow. The LV end-diastolic volume (LVEDV) and LV end-systolic volume (LVESV) were quantified by M-mode echocardiography and were corrected by two-dimensional guided biplanar Simpson’s method of discs for measurement [11, 12].

Atrial fibrillation was defined as paroxysmal if atrial fibrillation episode lasting for 7 days or less, persistent if continuous atrial fibrillation episode lasting for more than 7 days, and longstanding persistent if continuous atrial fibrillation episode lasting for more than 1 year.

### Study endpoints

The primary study endpoints were the recurrence of VT/VF and ICD therapy. The secondary endpoints were cardiovascular death (death related to heart failure and cardiac arrhythmia) and all-cause mortality (including sepsis, hepatic failure and brain hemorrhage).

### Statistical analysis

Data are presented as mean ± SD or percentage. The clinical characteristics (general demographics, underling heart diseases, comorbidities, functional class of heart failure, QRS duration, medications, LVEDV, LVESV, LV function, VT/VF detection zone and VT ablation) between the study groups were compared using *t*-test for continuous variables or chi-square test for categorical variables. Receiver operating characteristic curve analysis was used to calculate the area under the curve for the optimal volume of LVEDV, LVESV and QRS width in predicting the recurrence of VT/VF. Univariate and multivariate Cox regression analyses were performed to identify the significant predictors for the recurrence of VT/VF after implant. Each independent variable was based on previous studies and conventional risk factors, and was expressed as a hazard ratio with 95% confidence interval. The Kaplan-Meier method and log-rank test were used to compare the event-free survival of the recurrence of VT/VF during follow-up. Statistical analysis was performed using statistical software (SPSS for Windows, version 22). A two-sided *p* value of < 0.05 was considered statistically significant.

## Results

### Baseline characteristics of study patients

**Fig. 1:**
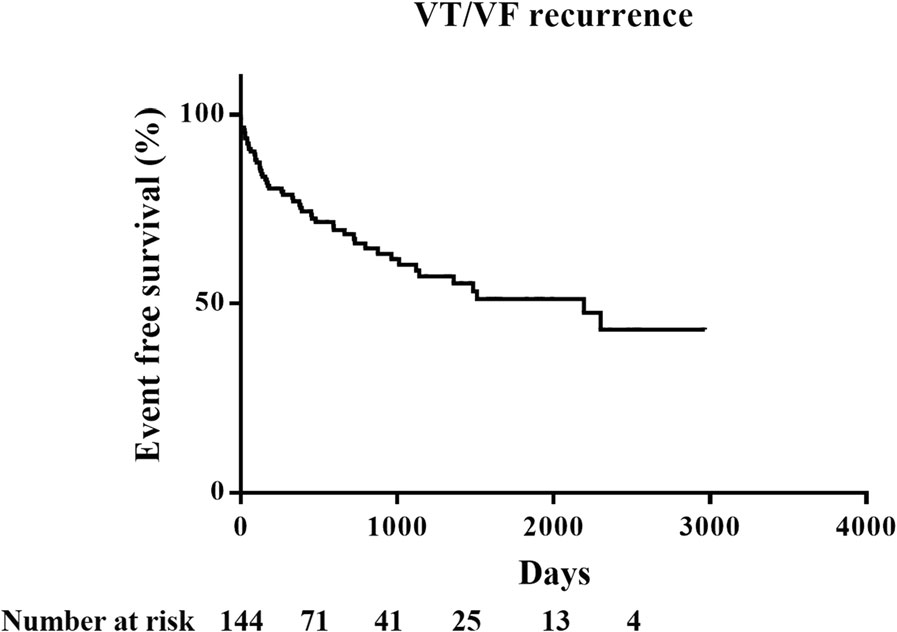
Kaplan-Meier plot proportion of patients free of recurrent ventricular tachycardia/ventricular fibrillation (VT/VF) events after implant in the study cohort

At a mean follow-up of 1110.5 ± 860.6 days, 53 patients (36.8%) had recurrence of VT/VF episodes and 91 patients had no recurrence of any VT/VF episode after ICD implant (Fig. 1). Table 1 lists the clinical characteristics of the study group. In this study, 71.5% of patients received renin-angiotensin-aldosterone antagonists and 60.4% of patients received ß–blocker therapy. The average age of patients in recurrent VT/VF group was 60 ± 13 years, and 77.4% of the patients were male. The average age of patients in no recurrent VT/VF group was 63 ± 14 years, and 80.2% of the patients were male. Patients with coronary artery disease in recurrent VT/VF group had higher prevalence of revascularization with coronary artery bypass graft surgery than patients with coronary artery disease in no recurrent VT/VF group. There was no significant difference in age, gender, body mass index, underling heart diseases, prevalence of comorbidities and atrial fibrillation, frequency of medications related to comorbidities and anti-arrhythmics, the average dosage of ß-blocker and anti-arrhythmics, QRS duration, the percentages of left bundle branch block and ventricular-dependent pacing, stage of LV diastolic dysfunction and heart failure status between the two groups. There was no significant difference in the prevalence of unstable VT (*p* = 0.590) and VT ablation plus medications (anti-arrhythmics and ß-blocker) (*p* = 0.358) between the two groups. Seventy nine patients received single chamber ICD and 65 patients received dual chamber ICD. There was no significant difference in the ICD type and the percentage of cardiac resynchronization therapy defibrillator recipients between the two groups. The lowest VT- and VF-detection rates were similar between two groups. Fifteen patients (10.4%) received catheter ablation and modification of arrhythmic substrates, including 6 patients (6.6%) in no recurrent VT/VF group, and 9 patients (17.0%) in recurrent VT/VF group (*p* = 0.087) (Table 1).

**Table 1:** Baseline characteristics of study patients

	No VT/VF group (*N* = 91)	Recurrent VT/VF group (*N* = 53)	*p* value
*General demographics*			
Age (year)	63 ± 14	60 ± 13	0.166
Male gender	73 (80.2)	41 (77.4)	0.419
BMI	25.5 ± 4.7	25.1 ± 3.8	0.686
*Heart disease*			
Congenital heart	4 (4.4)	0 (0)	0.297
CAD	51 (56.0)	29 (54.7)	0.979
Treatment in CAD patients			0.020
CABG	5 (9.8)	10 (34.5)	
PCI	41 (80.4)	18 (62.1)	
Medical treatment	5 (9.8)	1 (3.4)	
DCM	16 (17.6)	11 (20.8)	0.397
HOCM	4 (4.4)	2 (1.9)	0.391
Idiopathic VF	8 (8.8)	3 (5.7)	0.370
Brugada syndrome	3 (3.3)	3 (5.7)	0.388
Long QT syndrome	4 (4.4)	3 (3.8)	0.391
Valvular heart disease	8 (8.8)	8 (15.1)	0.187
*Comorbidity*			
Hypertension	52 (57.1)	26 (49.1)	0.222
Diabetes	24 (26.4)	20 (37.7)	0.108
Prior stroke	10 (11.0)	7 (13.2)	0.441
Hyperlipidemia	28 (30.8)	14 (26.4)	0.360
ESRD	6 (6.6)	5 (9.4)	0.377
*Atrial fibrillation* (%)			0.634
No	67 (73.6)	40 (75.5)	
Paroxysmal	17 (18.7)	11 (20.8)	
Persistent	7 (7.7)	2 (3.8)	
*Heart failure*			0.865
Without heart failure	29 (31.9)	16 (30.2)	
NYHA functional class I	16 (17.6)	11 (20.8)	
NYHA functional class II	27 (29.7)	13 (24.5)	
NYHA functional class III	14 (15.4)	8 (15.1)	
NYHA functional class IV	5 (5.5)	5 (9.4)	
*Diastolic function* (%)			0.322
Normal	11 (12.9)	11 (22.9)	
Stage I	40 (47.1)	24 (50.0)	
Stage II	14 (31.8)	8 (22.9)	
Stage III	5 (8.2)	5 (4.2)	
*QRS length (msec)*	115.3 ± 30.7	121.8 ± 32.3	0.231
*LBBB*	2 (2.2)	4 (7.5)	0.121
*Medications*			
ACEI/ARB	63 (69.2)	32 (60.4)	0.184
ß-blocker	59 (64.8)	28 (52.8)	0.107
Bisoprolol (the average dose) (mg)	3.5 ± 2.2	3.2 ± 1.5	0.530
Carvedilol (the average dose) (mg)	17.2 ± 10.0	9.1 ± 13.1	0.651
Diuretic	30 (33.0)	15 (28.3)	0.348
Statin	29 (31.9)	20 (37.7)	0.473
Spironolactone	11 (12.1)	11 (20.8)	0.125
Anti-platelet agent	54 (59.3)	29 (54.7)	0.604
Warfarin	12 (13.2)	9 (17.0)	0.626
NOAC	3 (3.3)	0 (0)	0.297
*Anti-arrhythmic medications*			
Amiodarone	62 (68.1)	33 (62.3)	0.295
The average dose (mg)	196.8 ± 85.4	214.3 ± 103.3	0.371
Dronedarone	2 (2.2)	0 (0)	0.532
The average dose (mg)	800		
Quinidine	2 (2.2)	0 (0)	0.532
The average dose (mg)	600		
Mexiletine	2 (2.2)	0 (0)	0.532
The average dose (mg)	300		
Sotalol	1 (1.1)	0 (0)	1.000
The average dose (mg)	160		
*CRT-D*	1 (1.1)	3 (5.7)	0.108
*ICD chamber*			0.731
Single	51 (56.0)	28 (52.8)	
Dual	40 (44.0)	25 (47.2)	
*Ventricular-dependent pacing*	4 (4.4)	1 (1.9)	0.428
*Hemodynamic condition*			0.590
Stable	60 (65.9)	32 (60.4)	
Unstable	31 (34.1)	21 (39.6)	
*Lowest VT-detection zone (bpm)*	165.9 ± 13.2	165.4 ± 10.5	0.865
*Lowest VF-detection zone (bpm)*	209.2 ± 13.5	208.2 ± 15.8	0.773
*Post VT ablation* (%)	6 (6.6)	9 (17.0)	0.087
Success	3 (50)	3 (33.3)	0.518
Failure	3 (50)	6 (66.7)	
*Combination of VT ablation plus medications (anti-arrhythmics and ß-blocker)*	6 (6.6)	6 (11.3)	0.358
*LV systolic function*			
LVEF (%)	49.5 ± 16.7	44.6 ± 18.5	0.105
LVEDV (mL)	162.0 ± 68.1	186.4 ± 84.5	0.060
LVEDV > 163.5 mL	36 (40.0)	33 (62.3)	0.010
LVESV (mL)	88.2 ± 61.3	111.4 ± 81.6	0.055
*1-year CV mortality*	1 (1.1)	5 (9.4)	0.028
*1-year all-cause mortality*	5 (5.5)	5 (9.4)	0.499
*Follow-up time (days)*	1019.5 ± 791.4	1266.7 ± 1028.5	0.108

Data are expressed as mean ± SD or as number (percentage)

*Abbreviations: VT ventricular tachycardia, *VF* ventricular fibrillation, *BMI* body mass index, *CAD* coronary artery disease, *CABG* coronary artery bypass graft surgery, *PCI* percutaneous coronary intervention, *DCM* dilated cardiomyopathy, *HOCM* hypertrophic obstructive cardiomyopathy, *RVOT* right ventricular outflow tract, *ESRD* end stage renal disease, *LVEF* left ventricular ejection fraction, *LVEDV* left ventricular end diastolic volume, *LVESV* left ventricular end systolic volume, *NYHA* New York Heart Association, *LBBB* left bundle branch block, *NOAC* non-vitamin K oral anticoagulants, CRT-D, cardiac resynchronization therapy-defibrillator, *ACEI* angiotensin-converting-enzyme inhibitor, *ARB* angiotensin receptor blocker, *CV* cardiovascular

### Determinants of recurrence of VT/VF during follow-up

**Fig. 2:**
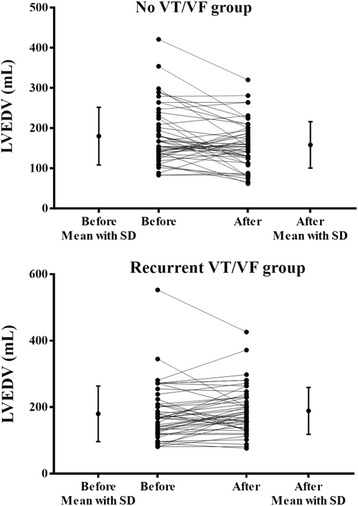
Changes in follow-up left ventricular end-diastolic volume (LVEDV) in the no ventricular tachycardia/ventricular fibrillation (VT/VF) recurrence group (*upper panel*) and the recurrent VT/VF group (*lower panel*). SD: standard deviation

Although there was no significant difference in LV ejection fraction between the two groups, LVEDV and LVESV were larger in patients with recurrent VT/VF than patients without recurrent VT/VF (Table 1). Receiver operating characteristic curve analysis was used to calculate the area under the curve for the optimal volume of LVEDV and LVESV in predicting the recurrence of VT/VF. Only LVEDV > 163.5 mL had significant predictive value for the recurrence of VT/VF (area under the curve: 0.602, *p* = 0.041). Moreover, the percentage of patients with LVEDV >163.5 mL was significantly higher in patients with recurrent VT/VF than patients without recurrent VT/VF (*p* = 0.010). Receiver operating characteristic curve analysis was also used to calculate the area under the curve for the optimal duration of QRS width in predicting the recurrence of VT/VF. QRS width over 125 msec had the best predictive value, although it did not reach statistical significance (area under the curve: 0.563, *p* = 0.209). Eighty-six patients (59.7%) had follow-up echocardiograms at a median follow-up period of 939.5 days. Notably, LVEDV at follow-up were significantly larger in patients with recurrent VT/VF than patients without recurrent VT/VF (191.00 ± 76.61 vs. 158.15 ± 57.82 mL, *p* = 0.028). Moreover, there was a trend, but not statistically significant that patients with no recurrent VT/VF were more likely to have reverse remodeling of LVEDV more prominently (−21.73 ± 54.49 vs. -0.74 ± 55.72 mL, *p* = 0.083) and more frequently (58.3% vs. 44.7%, *p* = 0.266) compared with patients with recurrent VT/VF (Fig. 2). The LV ejection fraction at follow-up remained similar between patients with recurrent VT/VF and patients without recurrent VT/VF (47.2 ± 16.3 vs. 46.7 ± 16.2%, *p* = 0.885).

**Fig. 3:**
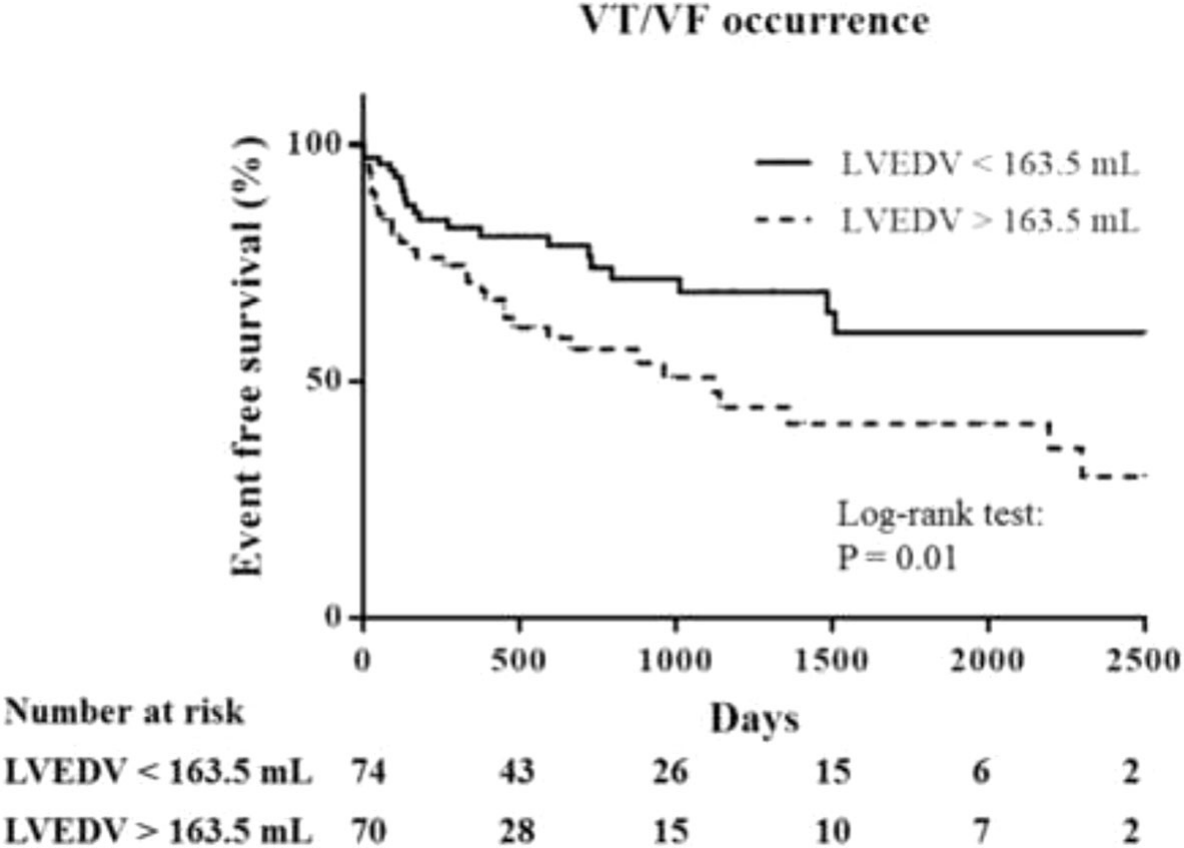
Kaplan-Meier plot proportion of patients free of recurrent ventricular tachycardia/ventricular fibrillation (VT/VF) events after implant stratified by left ventricular end-diastolic volume (LVEDV) of 163.5 mL

By univariate Cox regression analyses, LV ejection fraction ≤ 30% (*p* = 0.031), larger LVEDV (*p* = 0.028), LVEDV > 163.5 mL (*p* = 0.012), and larger LVESV (*p* = 0.025) were significant predictors for VT/VF recurrence during follow-up (Table 2). QRS width > 125 msec was also a significant predictor for VT/VF recurrence during follow-up (*p* = 0.049). There was a trend, but not statistically significant that anti-arrhythmics had a protective role for VT/VF recurrence (hazard ratio: 0.611, 95% confidence interval: 0.298 ~ 1.252, *p* = 0.178) (Table 2). However, only LVEDV > 163.5 mL (hazard ratio: 2.549, 95% confidence interval: 1.249 ~ 5.201, *p* = 0.010) and QRS width > 125 msec (hazard ratio: 2.173, 95% confidence interval: 1.030 ~ 4.586, *p* = 0.042) were independent predictors for recurrence of VT/VF during follow-up after multivariate adjustment (Table 2) (Fig. 3).

**Table 2:** Univariate and multivariate Cox regression analyses in terms of VT/VF recurrence

	Univariate analysis			Multivariate analysis		
Variables	Hazard ratio	95% CI	*p*-value	Hazard ratio	95% CI	*p*-value
Atrial fibrillation (paroxysmal and persistent)	1.095	0.584 ~ 2.052	0.777			
LVEF (%)	0.986	0.970 ~ 1.001	0.074			
LVEF ≦ 30%	1.995	1.064 ~ 3.740	0.031			
LVEDV (mL)	1.004	1.000 ~ 1.007	0.028			
LVEDV > 163.5 mL	2.042	1.170 ~ 3.562	0.012	2.549	1.249 ~ 5.201	0.010
LVESV (mL)	1.004	1.001 ~ 1.008	0.025			
Heart failure NYHA functional class ≧ 3	1.293	0.688 ~ 2.430	0.425			
QRS width (msec)	1.008	1.000 ~ 1.016	0.059			
QRS width > 125 msec	2.067	1.003 ~ 4.260	0.049	2.173	1.030 ~ 4.586	0.042
Ischemic cardiomyopathy	0.998	0.580 ~ 1.717	0.995			
Dilated cardiomyopathy	0.851	0.437 ~ 1.655	0.634			
Anti-arrhythmic drugs	0.611	0.298 ~ 1.252	0.178			
Post VT ablation	0.844	0.272 ~ 2.615	0.768			

*Abbreviations: VT ventricular tachycardia, *VF* ventricular fibrillation, *CI* confidence interval, *LVEF* left ventricular ejection fraction, *LVEDV* left ventricular end diastolic volume, *LVESV* left ventricular end systolic volume, *NYHA* New York Heart Association

### Cardiovascular death and all-cause mortality during follow-up

The average follow-up periods in the no VT/VF recurrence group and the recurrent VT/VF group were 1019.5 ± 791.4 days and 1266.7 ± 1028.5 days, respectively (*p* = 0.108). The 1-year cardiovascular mortality was significant higher in the recurrent VT/VF group than the no recurrence VT/VF group (9.4% vs. 1.1%, *p* = 0.028) (Table 1). However, there was no significant difference in the 1-year all-cause mortality between the two groups (9.4% vs. 5.5%, *p* = 0.499).

## Discussion

This study identifies and reports 36.8% of ICD recipients for secondary prevention having recurrent VT/VF at a mean follow-up of 1110.5 ± 860.6 days. LVEDV > 163.5 mL and QRS width > 125 msec were independent predictors for recurrence of VT/VF during follow-up after multivariable adjustment.

In the multicentre COMFORT trial, Zaman et al. reported that secondary prevention ICD recipients had more recurrent VT/VF than primary prevention ICD recipients (37.1% vs. 19.9% at 2-year follow-up) [13]. In our study, 36.8% of ICD recipients for secondary prevention had recurrent VT/VF, which was comparable to the recurrent rate reported in the COMFORT trial.

Ventricular tachyarrhythmia is an important cause of cardiovascular mortality, morbidity and heart failure hospitalization in a wide variety of heart diseases [14]. Implantable cardioverter defibrillator is now widely used in patients who survive sustained VT or VF, or who are at high risk for sudden cardiac death [1, 15]. Although ICDs can decrease risk of arrhythmic death, long-term total mortality still depends on the severity of the underlying heart diseases and associated comorbidities with the increase in nonarrhythmic deaths completely offsetting the decrease in arrhythmic deaths [14]. In our study, cardiovascular mortality was significant higher in the recurrent VT/VF group than the no recurrence VT/VF group, whereas there was no difference in the all-cause mortality (including nonarrhythmic deaths) between the two groups.

According to current guideline, LV ejection fraction has been selected as the most important criteria for ICD implantation in primary and secondary prevention settings [1]. However, several studies have shown improvement of LV ejection fraction during follow-up in primary prevention ICD recipients [16, 17]. Notably, Kini et al. reported that approximately 25% of primary prevention ICD recipients may no longer meet guideline indications for ICD use at the time of generator replacement due to improvement of LV ejection fraction, and these ICD recipients received subsequent ICD therapies at a significantly lower rate [16]. Additionally, in MADIT-CRT trial, patients who achieve LV ejection fraction normalization (>50%) during follow-up have very low absolute and relative risk of ventricular tachyarrhythmias and a favorable clinical course within 2.2 years of follow-up, and downgrade from cardiac resynchronization therapy-defibrillator to cardiac resynchronization therapy-pacemaker at the time of battery depletion for cost savings could be considered in these patients if no ventricular tachyarrhythmias have recurred [17]. However, ICD therapy is reimbursed only for secondary prevention in many countries, and limited data regarding LV remodeling as predictor for recurrent VT/VF are available for secondary prevention ICD recipients. Klein et al. reported that LV ejection fraction < 40%, permanent atrial fibrillation and QRS duration > 150 msec are independent predictors for VT/VF recurrence in ICD recipients predominantly for secondary prevention [8]. However, Freedberg et al. reported that LV ejection fraction was not a predictor for subsequent ICD therapy in secondary prevention ICD recipients [9]. In our study for secondary prevention ICD recipients, LVEDV > 163.5 mL and QRS width > 125 msec were independent predictors for recurrence of VT/VF during follow-up after multivariable adjustment (including LV ejection fraction, atrial fibrillation and heart failure status), and LVEF ≤ 30% was no longer a predictor after multivariable adjustment (Table 2).

### Study limitations

There are several limitations in this study. Firstly, this retrospective analysis bears the inherent limitations of these types of studies. Secondly, LVEDVs were estimated by M-mode echocardiography and were corrected by two-dimensional guided biplanar Simpson’s method of discs for measurement and this quantified method has been proven to be an acceptable method to evaluate LV volume. Moreover, there was a trend that patients with no recurrent VT/VF were more likely to have reverse remodeling of LVEDV during follow-up compared with patients with recurrent VT/VF (Fig. 2).

## Conclusions

LV remodeling and QRS width > 125 msec were independent predictors for recurrence of VT/VF during follow-up in secondary prevention ICD recipients under optimal medical therapy, independent of LV ejection fraction.

## Abbreviations

ICD: Implantable cardioverter defibrillator
LV: Left ventricular
LVEDV: LV end-diastolic volume
LVESV: LV end-systolic volume
VT/VF: Ventricular tachycardia/Ventricular fibrillation
